# Effect of corpus callosum agenesis on the language network in children and adolescents

**DOI:** 10.1007/s00429-020-02203-6

**Published:** 2021-01-26

**Authors:** Lisa Bartha-Doering, Ernst Schwartz, Kathrin Kollndorfer, Florian Ph. S. Fischmeister, Astrid Novak, Georg Langs, Harald Werneck, Daniela Prayer, Rainer Seidl, Gregor Kasprian

**Affiliations:** 1 Department of Pediatrics and Adolescent Medicine, Medical University of Vienna, Waehringer Guertel 18-20, 1090 Vienna, Austria; 2 Comprehensive Center for Pediatrics, Medical University of Vienna, Vienna, Austria; 3 Department of Biomedical Imaging and Image-Guided Therapy, Medical University of Vienna, Vienna, Austria; 4 Institute of Psychology, University of Graz, Graz, Austria; 5 Department for Psychology of Development and Education, University of Vienna, Vienna, Austria

**Keywords:** Corpus callosum, Corpus callosum agenesis, Language network, Functional connectivity, Language abilities

## Abstract

The present study is interested in the role of the corpus callosum in the development of the language network. We, therefore, investigated language abilities and the language network using task-based fMRI in three cases of complete agenesis of the corpus callosum (ACC), three cases of partial ACC and six controls. Although the children with complete ACC revealed impaired functions in specific language domains, no child with partial ACC showed a test score below average. As a group, ACC children performed significantly worse than healthy controls in verbal fluency and naming. Furthermore, whole-brain ROI-to-ROI connectivity analyses revealed reduced intrahemispheric and right intrahemispheric functional connectivity in ACC patients as compared to controls. In addition, stronger functional connectivity between left and right temporal areas was associated with better language abilities in the ACC group. In healthy controls, no association between language abilities and connectivity was found. Our results show that ACC is associated not only with less interhemispheric, but also with less right intrahemispheric language network connectivity in line with reduced verbal abilities. The present study, thus, supports the excitatory role of the corpus callosum in functional language network connectivity and language abilities.

**Supplementary Information:**

The online version contains supplementary material available at 10.1007/s00429-020-02203-6.

## Introduction

The corpus callosum (CC) is involved in the neural organization of language. In a recent functional magnetic resonance imaging (fMRI) study in healthy children, we described the integrative function of the central and posterior parts of the CC in the language network allowing stronger interhemispheric functional connectivity and enhanced language abilities (Bartha-Doering et al. 2020). These findings are in line with the previous studies on the association of language lateralization and callosal measures, where weaker language lateralization was associated with increased posterior CC size and volume in healthy adults (Hines et al. 1992; Josse et al. 2008).

In this study, we ask the question if these findings can be transferred to patients with altered CC morphology. To the best of our knowledge, there are no language-specific functional connectivity studies available in such a patient population; however, some studies used functional magnetic resonance imaging (fMRI) in patients with disrupted or nonexistent callosal connections to investigate resting-state networks, i.e. regional interactions independent of a specific task condition. In patients after surgical callosotomy due to intractable epilepsy, studies have reported markedly reduced interhemispheric functional resting-state connectivity and, thus, strongly support a causal role of the CC in maintaining interhemispheric functional connectivity (Roland et al. 2017; Johnston et al. 2008). Agenesis of the corpus callosum (ACC), however, is quite a different condition where callosal interhemispheric connectivity does never or only partially develop. In the absence of the CC, evidence for white matter circuit reorganization has been given, allowing for some kind of interhemispheric transfer (Wahl et al. 2009; Jakab et al. 2015; Meoded et al. 2015). The Probst bundle, the sigmoid bundle and two aberrant midbrain and ventral forebrain tracts represent the main components of altered brain circuitry in individuals with ACC (Tovar-Moll et al. 2007). Accordingly, fMRI studies emphasize that functional coupling of both hemispheres within resting-state networks may be nearly normal in ACC patients (Tovar-Moll et al. 2007; Tyszka et al. 2011; Owen et al. 2013; Khanna et al. 2012). In line with this, an fMRI study on six adult patients with ACC did not find significant differences in language lateralization indices between ACC patients and six IQ-matched healthy controls (Pelletier et al. 2011). However, other studies found less language lateralization and/or increased right hemisphere activation in ACC patients as compared to controls (Komaba et al. 1998; Riecker et al. 2007; Hinkley et al. 2016).

Based on these studies, early compensation for the absence of the CC seems possible. This explains the frequently reported favourable cognitive development in children with isolated ACC with overall intellectual abilities often described within the normal to low average range (Moutard et al. 2012; Siffredi et al. 2013). However, when investigated in detail, patients with ACC often exhibit deficits in high-level cognitive functions, including slower reaction times and processing speed (Marco et al. 2012), abstract reasoning, and problem solving (Hinkley et al. 2012; Siffredi et al. 2013; Brown and Paul 2000). In basic language functioning, various subtle deficits have been described, most often occurring in phonological and syntactic processing (Tappe 1999; Dennis 1981; Temple et al. 1989, 1990; Sanders 1989). More profound impairments have been reported in paralinguistic functioning. Children and adults with isolated ACC show difficulties in understanding idioms, proverbs, affective prosody and narrative humour (Brown et al. 2005; Paul et al. 2003). Furthermore, individuals with ACC are reported to exhibit deficits in the expression of emotions and their conversational abilities are described as ‘meaningless’ and ‘out-of-place’ (O'Brien 1994). In addition, verbal memory functions, including encoding, retention and retrieval are reduced in many individuals with ACC (Erickson et al. 2014; Geffen et al. 1994).

The present study investigated, for the first time, a possible link between language abilities and connectivity within the functional language network in children with ACC and may thus further our understanding of the role of the CC during the development of the language network. This is of particular interest as the existence of compensatory commissural tracts did not yet prove to be a good predictor of cognitive outcome in CC agenesis (Hannay et al. 2009; Severino et al. 2017). The investigation of the early functional language network may help to predict cognitive development. We, therefore, investigated the language network using task-based fMRI and language abilities with a comprehensive neurolinguistic test battery in four cases of complete ACC, two cases of partial ACC and six matched healthy controls. We hypothesized that comparable to our findings in healthy children, fewer interhemispheric functional language network connectivity would be associated with weaker language functions in children with ACC.

## Methods

### Participants

Six children with ACC, aged 6 to 15, were recruited at the neuropediatric outpatient unit of the Department of Pediatrics and Adolescent Medicine, Medical University of Vienna. Three patients had complete ACC, three patients showed partial ACC, as diagnosed by MRI (Table 1). None of the patients had known chromosomal or genetic abnormalities or extracranial abnormalities. No patient suffered from epilepsy or was under anticonvulsant therapy.

**Table 1:** Demographics and MRI findings

Study participants	Age	Sex	Handedness EHI*	MRI findings
ACC patients				
1	10	m	− 1.00	Complete absence of the CC, Probst bundles structurally present, anterior commissure present, severe colpocephaly
2	9	m	− 1.00	Complete absence of the CC, anterior commissure thick and present, moderate colpocephaly, bilateral Probst bundles structurally present
3	12	m	0.70	Partial callosal agenesis, extremely shortened and thinned, Probst bundle structurally left larger than right
4	15	f	1.00	Partial callosal agenesis, extremely shortened, only anterior portion present (genu and truncus), fornix and hippocampal commissure present, normal anterior commissure
5	14	m	0.70	Partial callosal agenesis, missing splenium, normal rostrum and genu, thinned truncus, anterior commissure present
6	6	m	− 1.00	Complete absence of the CC, anterior commissure present, asymmetric colpocephaly right > left, associated malformation of cortical development left central region (schizencephaly), Probst bundles structurally present
Group means (SD)	11.00 (3.35)		− 0.10 (.99)	
Controls				
Group means (SD)	11.17 (2.56)		0.88 (0.19)	

*EHI *Edinburgh Handedness inventory; the scale of the EHI ranges from − 1 (completely left handed) to + 1 (completely right handed)

We furthermore included six healthy, right-handed children matched for sex and age in this study. The controls had no history of neurological or psychiatric disease nor any clinical evidence of neurological dysfunction or developmental delay. They were recruited by blackboard announcement and flyer distribution. Further inclusion criteria for all study participants were native German speaking, normal hearing, normal or corrected-to-normal vision and no MRI contraindications.

Study participants were investigated using structural and functional MRI as well as neurolinguistic assessment. All children received a 30 € voucher for a bookstore. The study was approved by the Ethics Committee of the Medical University of Vienna and in accordance with the Helsinki Declaration of 1975. For children, age-appropriate assent forms were provided, parents received a parental permission form. All children and one parent per child gave written, informed consent prior to inclusion.

Table 1 depicts demographic information of the individual study participants. Although all healthy controls were right handed, three of the ACC patients exhibited clear left handedness, as measured with the Edinburgh Handedness Inventory EHI (Oldfield 1971).

### Data acquisition

#### MRI image acquisition

All participants were scanned on a 3 T Siemens TIM Trio whole-body MR-Tomograph (Siemens Medical Solutions, Erlangen Germany) and equipped with a high-performance gradient system to support fast, high-resolution whole-brain echo-planar imaging. 3D structural MRI scans were performed using an isocubic magnetization-prepared rapid gradient-echo (MPRAGE, T1-weighted, TE/TR_4.21/2300 ms, inversion time 900, with a matrix size of 240 × 256 × 160, voxel size 1 × 1 × 1.10 mm, flip angle 9°) sequence. FMRI was acquired using a phase-corrected blipped gradient echo, single-shot echo-planar imaging (EPI) sequence. Altogether, 200 EPI volumes were acquired with a square FOV of 210 mm, voxel size 2.1 × 2.1 × 4 mm, 20 slices with a gap of 25 percent were aligned parallel to the AC–PC plane; repetition time (TR) was 2000 ms, echo time (TE) 42 ms and the flip angle was set to 90°.

#### FMRI Paradigm

The German version of an auditory description definition task adapted from Berl et al. (2014) and Sepeta et al. (2016) was administered during fMRI assessment. Detailed description of this paradigm can be found in Bartha-Doering et al. (2018a, b) or (2019). In the auditory description definition condition, the participants heard the definition of an object followed by a noun and were instructed to press a button each time the definition truly described the noun. The control condition consisted of reverse speech, with some items additionally containing a pure tone at the end. The participants were instructed to press the button each time they heard the tone. Three age-adjusted versions of the fMRI paradigm were available (7–9 years old, 10–12 years old, 13–16 years old). The total fMRI scan time was 6 min, 40 s.

#### Cognitive examinations

Standardized tests of language comprehension, naming and verbal fluency were used to assess verbal abilities with a particular focus on semantic language processing. *Language comprehension* was measured with the Token Test for Children (McGhee et al. 2007), where tokens varying in size and shape have to be moved according to auditory commands with increasing length and linguistic complexity. *Naming* was examined using the Wortschatz- und Wortfindungstest WWT (Glück 2011). The WWT provides information about expressive vocabulary in different lexical categories, including nouns, verbs, and adverbs/adjectives, and has no time limit. *Verbal fluency* was evaluated using the Regensburger Wortflüssigkeitstest (RWT) (Aschenbrenner et al. 2001), which requires the participant to name, within 2 min, as many words as possible of the semantic category animals.

### Data analysis

#### Preprocessing

The images were preprocessed using Statistical Parametric Mapping 12 software (Wellcome Department of Cognitive Neurology, London, UK) and the CONN toolbox 16b (Whitfield-Gabrieli and Nieto-Castanon 2012) working on MATLAB 2019a. EPI volumes were spatially realigned and corrected for movement. Frame to frame displacement between successive volumes was estimated by calculating the Euclidian distance from the translational parameters obtained from the realignment. Customized prior probability maps and a customized T1 template, matched to age and sex composition of the study group, were created by employing the Template-O-Matic (TOM) toolbox (Wilke et al. 2008). After co-registration, the derived spatial normalization parameters were used to normalize the functional volumes. Normalized EPI volumes were visually inspected for maximum overlap with the template and then smoothed using a spatial filter kernel of FWHM = 4 mm.

#### Analysis of the lateralization of language activation

BOLD signal increases pertaining to task-evoked responses in brain activity were modeled using a general linear model as implemented in SPM. A regressor modeling residual movement-related variance (translational and rotational movement) was included in the model as a covariate of no-interest. Language activation was measured by contrasting auditory description definition task condition > reversed language control condition. To examine the group effect of functional brain activations, fixed effects analyses were performed (*p*_FWE_ < 0.05, extend threshold *k* = 40 voxels) due to the small number of subjects. Thus, the fMRI results are valid only for the investigated group of subjects. Single-subject fMRI activations were analyzed with a significance threshold of *p*_uncorr_ < 0.001, extend threshold *k* = 100 voxels.

Individual lateralization of activations was estimated at the single-subject level by use of the LI-toolbox (Wilke and Lidzba 2007). LIs were computed for the whole brain. In order to avoid the threshold dependency of LIs, a bootstrapping approach was employed. LIs were calculated according to the formula (Σ activation left − Σ activation right)/(Σ activation left) + Σ activation right) where “Σ activation” is the sum of activated voxels. Based upon previous studies (Lidzba, Kupper, et al. 2017a, b; Bartha-Doering et al. 2018a, b), LI was categorized as left lateralized if ≥ 0.20, bilateral if within -0.20 and + 0.20 or right if ≤ − 0.20.

#### Task-based connectivity analysis

Functional connectivity is defined as the temporal coincidence of spatially distant neurophysiological events (Friston 2011). In the present study, two regions were considered to show functional connectivity if there was a statistical relationship between their measures of activity.

Functional connectivity analysis was carried out in the CONN toolbox using SPM preprocessed data. Additionally, a band‐pass filter (0.008–0.09 Hz) using SPM's Fast-Fourier-Transformation-based procedure for bandpass filtering was applied to the time series. ART-based outlier detection was performed (97th percentiles in normative sample, global-signal *z *value threshold of 5, subject-motion threshold 0.9 mm). Segmented white matter and cerebrospinal fluid were identified by CONN using the aCompCor method (Behzadi et al. 2007). Noise-related confounds, along with realignment parameters, were regressed from the data before calculating functional connectivity.

To assess task-related connectivity changes, we conducted a seed-based ROI-to-ROI analysis to create a connectivity map separately for both task conditions. A bivariate correlation was used to determine the associations between each of the ROI-to-ROI pairs; afterwards, these correlation coefficients were Fisher’s *z*-transformed and submitted to a 2 group × 2 condition linear model. Within this model, language-related connectivity changes between ACC patients and controls were described using a two-sample *t* tests comparing positive task differences in functional language network connectivity. This functional language network connectivity was obtained by directly contrasting the auditory description definition task condition with the reversed language control condition.

Second level regression analyses were employed to describe the effect of language scores on functional network connectivity separately per group. To this end, a linear model with individual language scores as covariates and the contrast between the two functional tasks (auditory description and reversed language control) was calculated. Within this model, separate regression analyses, one per score, were calculated and tested for significance. The significance level for all tests was set at *p*_FDR_ < 0.05.

To evaluate differences in language network connectivity between ACC patients and controls, a whole‐brain ROI-to-ROI functional connectivity analysis was carried out using the default atlas (132 ROIS) in the CONN toolbox that combines the FSL Harvard–Oxford atlas (Caviness et al. 1996) and the AAL atlas (Tzourio-Mazoyer et al. 2002). To evaluate the effect of the language scores on network connectivity, language ROIs were selected from the Brainnetome Atlas (Fan et al. 2016) that were characterized as involved in language processing, along with their contralateral homologues. The Brainnetome Atlas uses meta data labels of the BrainMap Database (www.brainmap.org/taxonomy) using forward and reverse inferences (Cieslik et al. 2013; Clos et al. 2013; Eickhoff et al. 2011). For our language nodes, we included regions that were involved in paradigms of speech, semantics, syntax and phonology, while we excluded regions that were only involved in orthography. In addition, we included the hippocampi and parahippocampal gyri within both hemispheres as their involvement in semantic language processing was indicated in the previous research (Bartha-Doering et al. 2018a, b; Bartha et al. 2003, 2005). In sum, we obtained a total of 60 language ROIs (please see Supplementary Table S1 for a list of all language ROIs).

#### Cognitive test analyses

Statistical analyses were conducted using IBM SPSS Statistics (Version 26). Raw scores of language tests were transformed into age-adjusted *z* scores for each test. For the WWT norms were only available until 11 years of age. Therefore, we transformed the WWT raw scores of the children aged 12–15 (*n* = 6) into *z* scores based on the 11-year-old children with the risk of an overestimation of WWT results in these participants. In line with clinical conventions, individual *z *scores from − 1 to 1 were defined within the average range. Performance below − 1 was read as below average and performance below − 2 was interpreted as reduced. To reduce the number of language variables for connectivity analyses, a mean *z *score was calculated from all language tests for each participant and used as “overall language score” in functional connectivity regression analyses.

As cognitive data were not normally distributed, group differences in cognitive test results were investigated by Mann–Whitney *U* test. Significance of findings was set based on a strict Bonferroni correction factor, i.e. *α* = 0.05/number of comparisons.

## Results

### Language abilities

**Fig. 1:**
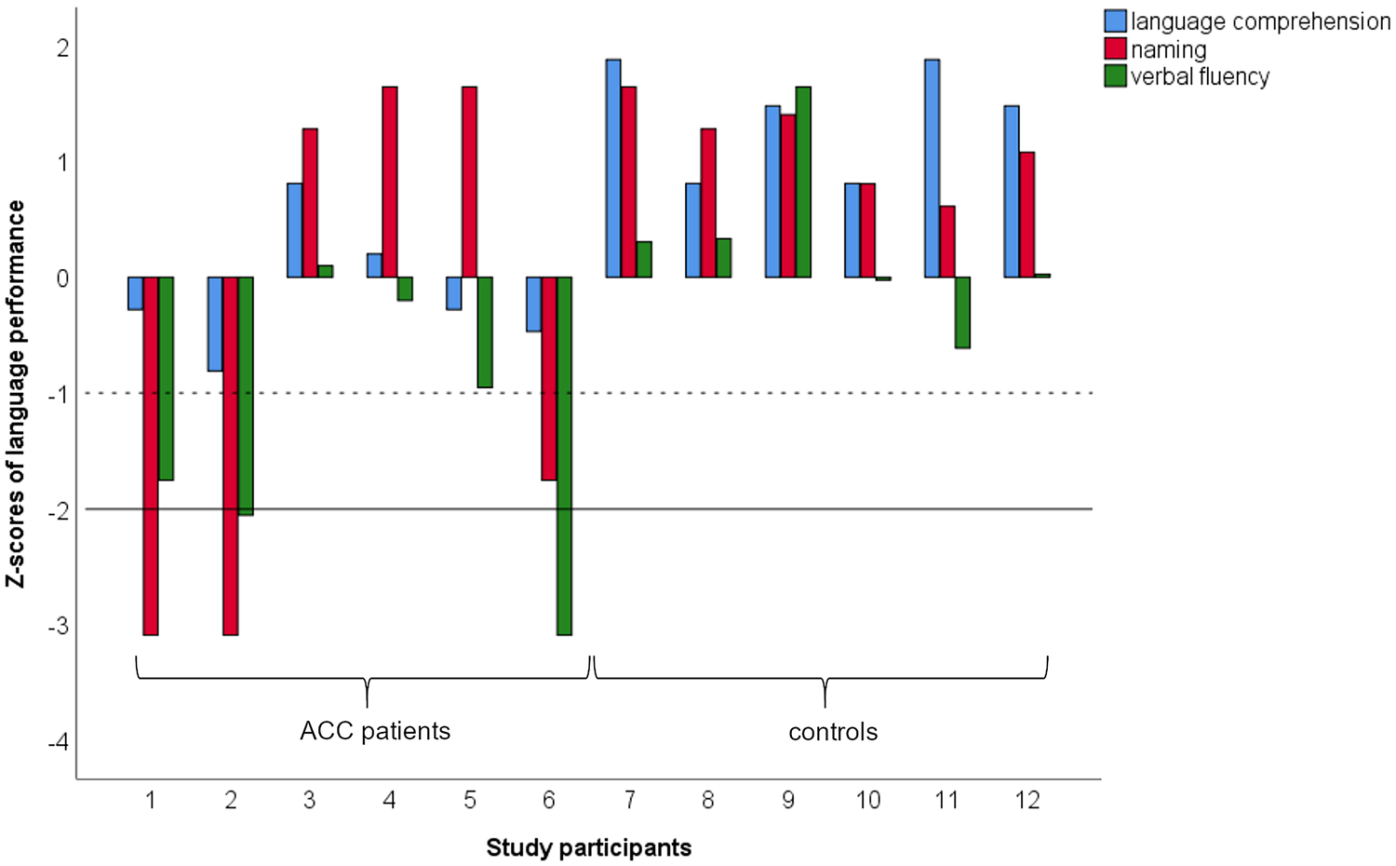
Individual language profiles in study participants. The solid line represents *z *scores—2 (impaired function), the dashed line *z* scores—1 (below average function)

Figure 1 displays the individual language profiles in study participants. Language scores were within, or above, normal limits for all control participants and participants with partial ACC. The three participants with complete ACC performed within normal limits on language comprehension, but were impaired on verbal fluency and/or naming. Figure 1 displays the individual language profiles in study participants (for more information on individual results, please see the Supplementary File, Table S2). Group comparisons are reported in Table 2.

**Table 2:** Differences in language measures between groups

	ACC patients	Controls	*U*	*p*	*r*
Language abilities	*z *scores mean, SD (range)	*z *scores mean, SD (range)			
Language comprehension	− 0.14, 0.57 (− 0.81 to 0.81)	1.39, 0.48 (0.81 to 1.88)	**1.0**	0**.004**	0**.80**
Naming	− 0.56, 2.34 (− 3.09 to 1.64)	1.14, 0.38 (0.61 to 1.64)	14.5	0.589	0.16
Verbal fluency	− 1.33, 1.20 (− 3.09 to 0.10)	2.78, 0.75 (− 0.61 to 1.64)	**4.0**	0**.026**	0**.65**
Language lateralization	LI scores mean, SD (range)	LI scores mean, SD (range)			
LI	0.22, 0.43 (− 0.50 to 0.60)	0.69, 0.12 (0.50 to 0.84)	**2.0**	0**.009**	0**.74**

Statistical significance after Bonferroni correction is indicated in bold

*LI *laterality index

### In-scanner task performances

In ACC patients, mean correct response to in-scanner tasks was 89.5% (SD 7.45), controls had a mean correct task performance of 93.0% (SD 7.24). Overall, these data indicate good task performances. In-scanner task performances did not significantly differ between groups [*U* = 13.0, *p* = 0.485, *r* = 0.23].

### Localization and lateralization of language activations

Head movement was within the tolerable limit in all children (overall movement group mean 0.19 mm, SD 0.32, range 0.03–1.21 mm) and did not significantly differ between groups, though it was larger in ACC patients than controls [*U* = 9.0, *p* = 0.180, *r* = 0.42]. In the individual analyses, two ACC patients revealed atypical language lateralization, both of them having a complete ACC (individual fMRI results and LIs are presented in the Supplementary file, Table S2). The other four ACC patients (1 complete, 3 partial ACC), as well as all healthy controls, showed left lateralized LIs. As a group, ACC patients exhibited significantly less left lateralized language than controls [*U* = 2.0, *p* = 0.009, *r* = 0.74] (Table 2).

### Whole-brain language network connectivity in ACC

**Fig. 2:**
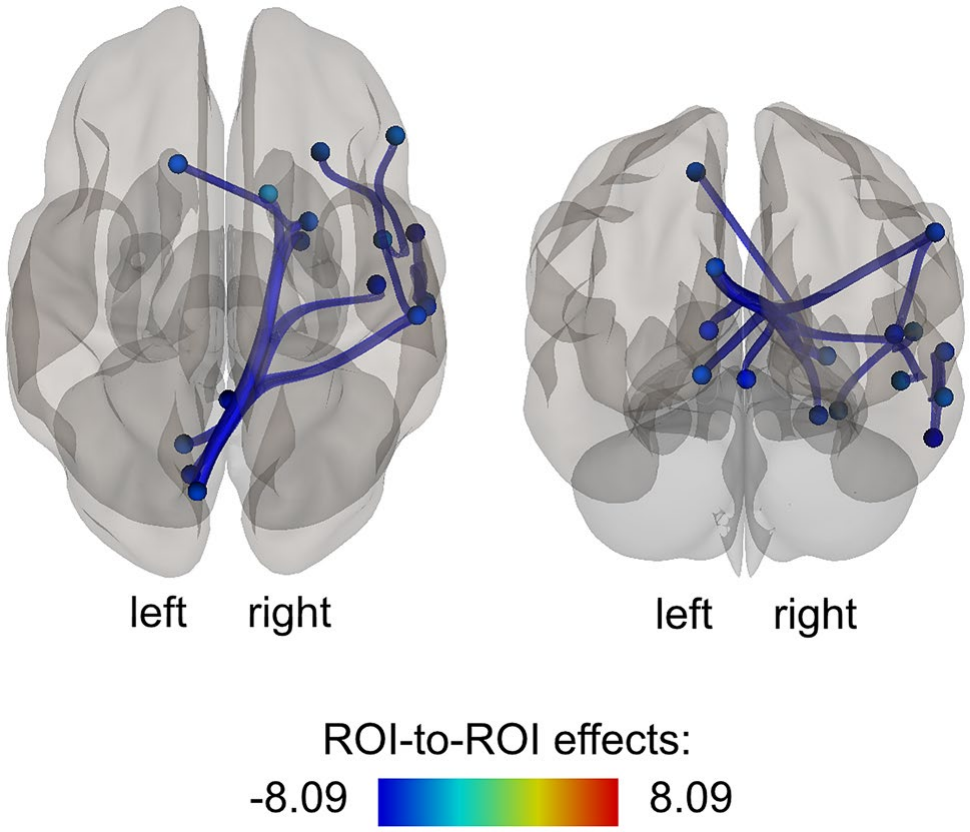
Contrast of whole-brain functional language network connectivity of ACC patients versus controls. Blue lines indicate decreased ROI-to-ROI functional connectivity. ACC patients showed a significant decrease in interhemispheric and right intrahemispheric functional connectivity as compared to controls. No functional network increase was observed in ACC patients versus controls

ROI-to-ROI analyses within the whole brain revealed significantly less functional language network connectivity in ACC patients as compared to controls (Table 3; Fig. 2). Interhemispheric functional connectivity was reduced from left mesial frontal and mesial occipital regions to the right supramarginal gyrus and to right basal ganglia and amygdala. Furthermore, within the right hemisphere, less functional connectivity was found within fronto-temporal regions and from Heschl`s gyrus to the vermis. In contrast, within the left hemisphere, ACC patients did not show less connectivity as compared to controls. Furthermore, functional language network connectivity was not increased in ACC patients compared to controls in any region.

**Table 3:** Functional language network connectivity contrasts between ACC patients and controls

ROI to ROI connectivity ACC patients > controls	*t *value	*p*_FDR_
Interhemispheric network		
L lingual gyrus—R supramarginal gyrus, ant	− 8.09	0.001
L intracalcerine cortex—R supramarginal gyrus, ant	− 5.41	0.019
L superior frontal gyrus—R putamen	− 5.81	0.023
L cuneal cortex—R putamen	− 4.90	0.039
L cuneal cortex—R amygdala	− 4.90	0.039
L cuneal cortex—R caudate	− 4.66	0.039
Left hemisphere network	ns	
Right hemisphere network		
R superior temporal gyrus, post—R middle temporal gyrus, ant	− 5.55	0.020
R superior temporal gyrus, post—R middle temporal gyrus, post	− 5.39	0.020
R Heschl’s gyrus—vermis	− 5.59	0.030
R inferior frontal gyrus, pars triangularis—R planum polare	− 5.59	0.030
R middle temporal gyrus, ant—R superior temporal gyrus, post	− 5.55	0.032
R supramarginal gyrus, ant—R frontal orbital cortex	− 4.52	0.048

*L * left hemisphere, *R * right hemisphere, *ant *anterior part, *post *posterior part

In sum, ACC patients revealed significantly reduced inter- and right intrahemispheric functional language network connectivity as compared to controls.

### The association of functional language network connectivity and language abilities

**Fig. 3:**
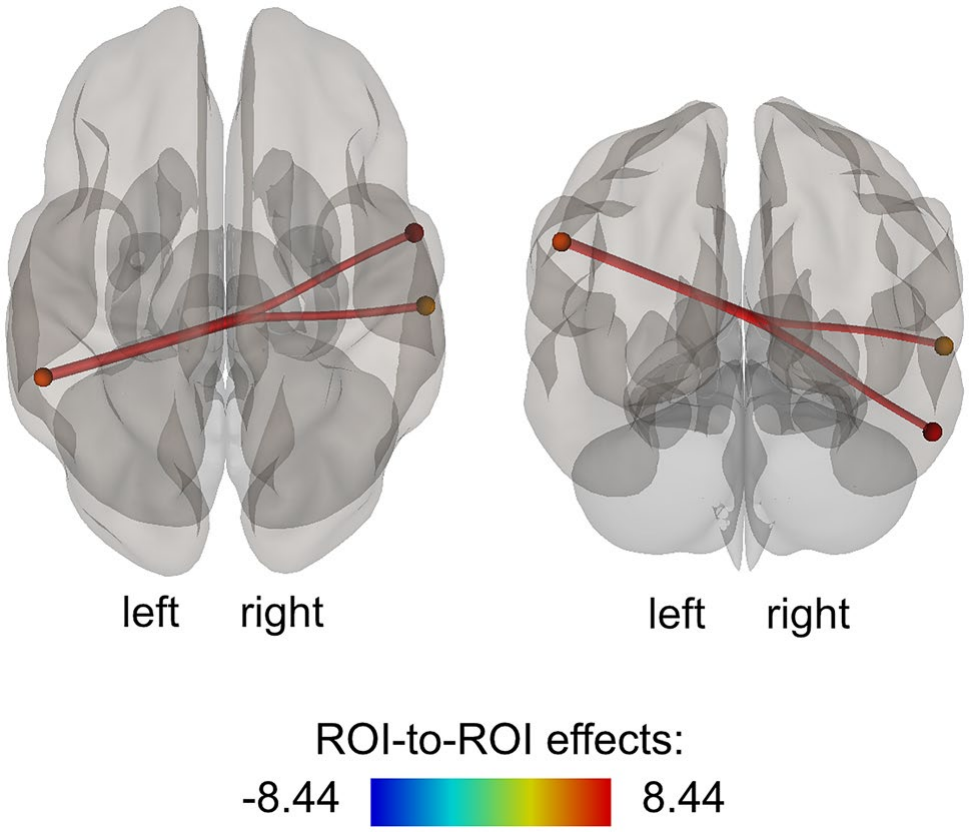
Associations of language abilities with functional language network connectivity within the ACC group. Red lines indicate increased ROI-to-ROI functional connectivity. Better overall language scores correlated with increased interhemispheric network connectivity. No functional network decrease was observed in association with better language functions

In ACC patients, second level regression analyses revealed a better overall language score being associated with stronger functional connectivity between the left supramarginal gyrus and the right superior and middle temporal gyri (Table 4; Fig. 3). The overall language score was not associated with any reduction of functional connectivity in ACC patients. In healthy controls, better language abilities were associated with stronger connectivity within the right hemisphere from the supramarginal to the fusiform gyrus, but the strength of the relationship between functional connectivity and language performance was smaller and did not reach significance (*t *test = 8.83, *p*_FDR_ = 0.06).

**Table 4:** Association of functional language network connectivity with language abilities

ROI to ROI connectivity associated with a better overall language score	*t *value	*p*_FDR_
ACC patients		
Functional connectivity increases		
L supramarginal gyrus, post—R superior temporal gyrus, post	8.44	0.038
L supramarginal gyrus, post—R middle temporal gyrus, ant	8.04	0.038
Functional connectivity decreases	ns	
Controls	ns	

*L *left hemisphere, *R *right hemisphere, *ant *anterior part, *post *posterior part

In sum, in ACC patients, stronger interhemispheric functional connectivity between temporal language areas was associated with better language abilities. In contrast, controls did not show a significant effect of language abilities on functional connectivity.

## Discussion

The aim of the present study was to investigate the functional organization of the language network in a case series of six patients with ACC. Those children with complete ACC presented impaired language functions, whereas children with partial ACC had intact verbal abilities. As a group, the ACC children revealed significantly worse verbal fluency and naming as compared to the healthy control group. Task-based functional connectivity analysis furthermore exhibited reduced interhemispheric and right intrahemispheric language network connectivity in children with ACC as compared to healthy controls. In the patient group, stronger interhemispheric functional connectivity was correlated with better language abilities, while controls did not show a significant association between language performance and connectivity.

### Interhemispheric connectivity in ACC

The findings of reduced interhemispheric functional language network connectivity in ACC underline the important role of the CC in the integration of linguistic information from both hemispheres. When compared with controls, ACC patients showed significantly less interhemispheric connectivity from left mesial frontal and mesial occipital regions to right temporal and subcortical areas. Interestingly, stronger interhemispheric connectivity was associated with better overall language performance in children with ACC. In healthy controls, the correlation of the relationship between language score and connectivity was smaller and did not reach significance. The findings in our ACC patients suggest that the existence of white matter tracts enabling some kind of interhemispheric functional transfer is favourable for language outcome in these children. This interhemispheric connectivity may be supported by a partially developed CC. Indeed, in our study, the three children with partial ACC performed better in the language tests than the three children with complete ACC. In the complete absence of CC, white matter reorganization may compensate for the lack of callosal connections and allow some kind of interhemispheric transfer. In fact, an enlarged anterior commissure has shown to be favourable for the interhemisperic integration of visual stimuli in patients with ACC (van Meer et al. 2016; Barr and Corballis 2002). In addition, the sigmoid bundle has been identified as an asymmetric, heterotopic commissural tract. Together with the Probst bundle and two novel aberrant midbrain and ventral forebrain tracts, it forms the fundamental brain circuitry in ACC patients (Tovar-Moll et al. 2014, 2007).

Nevertheless, those routes may be functionally costly and less effective (Ocklenburg et al. 2015). Although resting-state networks have often been found bilaterally symmetrical in ACC (Tyszka et al. 2011; Owen et al. 2013), increased task demands during fMRI measurements revealed specific functional connectivity deficits in ACC patients (Hearne et al. 2019). Accordingly, studies showed that individuals with ACC primarily exhibit deficits in higher level cognitive functions, including problem solving and processing speed (Siffredi et al. 2013). Similarly, linguistic deficits in our sample were only found in verbal fluency and naming, whereas language comprehension was intact in all children. Thus, verbal abilities with a higher processing load were more severely impaired.

The findings in ACC patients are in line with previous research in healthy children and adolescents, showing that bilateral language representation is favourable for language abilities (Bartha-Doering et al. 2018a). The present study furthermore resumes a recent study from our lab that demonstrated the integrative function of the posterior CC in functional language network connectivity, fostering improved interhemispheric functional connectivity and enhanced language abilities in healthy children (Bartha-Doering et al. 2020). Interestingly, some previous studies point to different effects of the CC on language lateralization in temporal and frontal brain areas dependent on the nature of task. Whereas for perceptual language tasks, the CC seems to play a predominantly excitatory role integrating posterior brain regions, some studies suggest an inhibitory role for the CC during language production in anterior brain areas (Josse et al. 2008; Thiel et al. 2006; Hines et al. 1992). This dichotomy of anterior and posterior language lateralization corresponds to the hypothesis that hemispheric specialization for language is not a uniform phenomenon but has complex task and region dependent characteristics (Josse and Tzourio-Mazoyer 2004; Cohen and Dehaene 2004; Boles et al. 2008; Piervincenzi et al. 2016).

### Intrahemispheric connectivity in ACC

Remarkably, ACC children showed not only less interhemispheric connectivity, this group of children also exhibited reduced right intrahemispheric language network connectivity. Reduced connectivity was found within temporal areas, between frontal and temporal regions and between the Heschl’s gyrus and the vermis. Interestingly, functional connectivity within the left hemisphere did not differ in ACC patients compared to healthy controls.

Previous diffusion tensor imaging studies in ACC have shown diffusion abnormalities and reduced volumes in white matter bundles not only between but also within the hemispheres (Nakata et al. 2009). Our results, thus, further emphasize the concept that ACC cannot be considered as the simple absence of the CC, but represents a condition associated with a globally altered structural brain organization and the inherent potential to (at least) partially establish normal function (Hinkley et al. 2012). Moreover, our results may also be interpreted considering the predominantly excitatory role of the CC in language network connectivity (Galaburda et al. 1990; Gazzaniga 2000). Reduced interhemispheric connectivity may also lessen stimulation of intrahemispheric language processes, resulting in weaker language abilities. This explanation is supported by the fact that we only observed less intrahemispheric functional language connectivity in ACC as compared to healthy controls, whereas ACC did not result in increased functional connectivity within one hemisphere. This result is in contrast to previous studies investigating resting-state networks in both humans and monkeys with split-brain conditions (Roland et al. 2017; O'Reilly et al. 2013; Johnston et al. 2008). In these studies, reduced inter- and increased intrahemispheric resting-state connectivity after callosotomy pointed to both excitatory (interhemispheric) and inhibitory (intrahemispheric) functions of the CC. However, callosotomy is quite a different condition compared to ACC and an increase of intrahemispheric functional connectivity found the day after callosotomy in some patients (Roland et al. 2017) should be interpreted with caution as a sign of functional reorganization (Mancuso et al. 2019a, b). Moreover, functional connectivity during resting state is different to task-based connectivity, as the covariance between ROIs during specific tasks reflects the degree to which two regions are coordinated in their specific activity, rather than a general shared co-activation (Tran et al. 2018), and the function of the CC may not be the same in different cognitive domains. Furthermore, it might be hypothesized that the role of the CC in functional connectivity is not only domain- but also demand-specific, an assumption supported by previous fMRI studies that have proven a positive correlation between task difficulty and bilateral activation volume (Caplan et al. 2002; Just et al. 1996; Just and Varma 2007; Kaan and Swaab 2002). Hence, the interaction between hemispheres may be especially beneficial under conditions of high complexity and attentional demand (Banich and Brown 2000). The present study, thus, supports the excitatory model proposing CC as the pathway that integrates information from both cerebral hemispheres, allowing improved inter- and intrahemispheric connectivity and better language functioning (Galaburda, Rosen, and Sherman 1990; Gazzaniga 2000).

### Hemispheric specialization

Although language is predominantly processed in the left hemisphere in most healthy children (Szaflarski et al. 2002, 2012), stronger functional connectivity with the right hemisphere enables better verbal abilities (Bartha-Doering et al. 2020). Thus, the CC seems to play an excitatory role in the integration of information of both hemispheres and language abilities profit from additional right hemisphere language processing that support and interact with left hemisphere processing. In healthy right-handed children, these language regions to the right seem to play a subordinate role; however, the situation might be different in children with ACC. In fact, two of our ACC children present atypical language lateralization, an observation in line with previous studies reporting a higher incidence of atypical language lateralization in ACC (Hinkley et al. 2012; Siffredi et al. 2013; Sauerwein and Lassonde 1994). These findings can be interpreted in terms of hemispheric autonomy in ACC: Interhemispheric exchange is diminished, hemispheric specialization is reduced, and both hemispheres are able to process specific cognitive demands to similar degrees (Ocklenburg et al. 2015), though often with less efficiency.

Our small ACC population includes three children with left handedness, two of them also presenting atypical language lateralization. The high proportion of left-handers in our study is consistent with the previous ACC studies that reported left handedness in 24% to 56% of their study populations (Siffredi et al. 2018; Labadi and Beke 2017; Sauerwein and Lassonde 1994; Chiarello 1980) and thus representative for the ACC population. The finding of atypical language lateralization in two of our six ACC children may be explained by their left handedness, as left handedness is associated with a higher increase of right hemisphere involvement in language processing (Carey and Johnstone 2014; Tzourio et al. 1998; Knecht et al. 2000). However, healthy left-handers also reveal a stronger functional connectivity between left and right language areas (Wiberg et al. 2019). Thus, the reduction of interhemispheric and right intrahemispheric language network connectivity in our ACC patients can not be explained by the increased left handedness in our ACC population. Rather, left handedness and reduced language network connectivity may be significantly but independently associated with ACC.

### Does functional connectivity reflect structural connectivity?

The present study found differences in functional interhemispheric connectivity between ACC patients and healthy controls that also affect heterotopic areas. At first glance these results do not fit to the well-established belief that callosal axons mainly connect homotopic cortices (Schmahmann and Pandya 2006). However, heterotopic transcallosal projections exist (Chovsepian et al. 2017; Hedreen and Yin 1981; Mancuso, Costa, et al. 2019a, b), especially in partial ACC. Wahl et al. (2009) investigated interhemispheric white matter connectivity in parietal ACC and identified not only homotopic but also heterotopic connections in the majority of their patients. Furthermore, the nature of homotopic and heterotopic connectivity varied considerably in their patient group.

Above all, functional connectivity does not imply any causal relationship and does not have to reflect a direct connection between functionally coupled areas. Rather, the correlation of two regions may be mediated via additional structures relaying information from one region to another (Damoiseaux and Greicius 2009). Contrary to structural connectivity, functional correlation of areas of interest can furthermore inform about increases and decreases of their functional connectivity and can thus provide additional information of the nature of their connectivity (Fox et al. 2005). A number of recent studies have revealed that functional connectivity strength correlates with structural connectivity strength (Damoiseaux and Greicius 2009), but there is not a direct one-to-one mapping between them, and functional networks often exceed patterns of structural connectivity (Adachi et al. 2012).

### Limitations

We transformed the WWT raw scores of the elder participants into *z* scores based on the 11-year-old children as there are no normative data for children older than 11 years of age. The mean difficulty to name the items of the WWT decreases exponentially and phases out in a flat curve with 10 years of age (Glück 2011); however, the risk remains that we overestimated *z *score results for the elder study participants. Thus, the fact that we did not find an association between naming performance and language connectivity in our study may also be due to a *z-*score transformation error in the older participants.

A further limitation of the present study is the presence of a small schizencephalic defect in the left central region in one ACC patient. This cortical malformation may have had impact on the functional network connectivity in this patient and may thus reduce the generalizability of findings.

Furthermore, as we administered only one fMRI run per participant, within-subject reliability of connectivity and LI could not be assessed.

Electrophysiological studies indicate that the information flow between language-relevant brain areas may depend on the contributions of distinct brain rhythms and point to the interplay of rapid excitation and slow inhibition that might be important for this interhemispheric communication (Steinmann and Gutschalk 2011; Schoffelen et al. 2017). FMRI measures haemodynamic response as the indirect consequence of neural activity and offers high spatial resolution, but cannot measure rapidly fluctuating brain activity. Identifying network interactions from the complementary haemodynamic and electrophysiological signals may help to explain these complex interactions between brain areas (Lei et al. 2011; Mulert 2013), thus, future studies may use simultaneous EEG-fMRI to offer new insights into the connectivity mechanisms in ACC.

### Conclusion

ACC is associated not only with a reduction of inter-, but also right intrahemispheric language network connectivity, going along with reduced verbal abilities. The present study, thus, supports the excitatory role of the CC in functional language network connectivity and language abilities.

## Supplementary Information

Below is the link to the electronic supplementary material.

Supplementary file1 (DOCX 836 KB)

## Data Availability

For reasons of data security, we are not able to provide original data and material of the study.
