# Supplementary material for: Effect of corpus callosum agenesis on the language network in children and adolescents
Source: Brain Struct Funct. 2021 Jan 26;226(3):701–13. doi: 10.1007/s00429-020-02203-6 (PMC7981296; doi:10.1007/s00429-020-02203-6)
Supplement: Supplementary file 1 — Supplementary file1 (DOCX 836 KB) [file 429_2020_2203_MOESM1_ESM.docx]

**Supplementary Information**

Table S1. Language ROIs

|  | Frontal Pole |
| --- | --- |
|  | Insular Cortex |
|  | Superior Frontal Gyrus |
|  | Middle Frontal Gyrus |
|  | Inferior Frontal Gyrus, pars triangularis |
|  | Inferior Frontal Gyrus, pars opercularis |
|  | Precentral Gyrus |
|  | Temporal Pole |
|  | Superior Temporal Gyrus, anterior division |
|  | Superior Temporal Gyrus, posterior division |
|  | Middle Temporal Gyrus, anterior division |
|  | Middle Temporal Gyrus, posterior division |
|  | Middle Temporal Gyrus, temporooccipital part |
|  | Inferior Temporal Gyrus, anterior division |
|  | Inferior Temporal Gyrus, posterior division |
|  | Inferior Temporal Gyrus, temporooccipital part |
|  | Supramarginal Gyrus, anterior division |
|  | Supramarginal Gyrus, posterior division |
|  | Angular Gyrus |
|  | Lateral Occipital Cortex, inferior division |
|  | Frontal Orbital Cortex |
|  | Parahippocampal Gyrus, anterior division |
|  | Parahippocampal Gyrus, posterior division |
|  | Lingual Gyrus |
|  | Temporal Fusiform Cortex, anterior division |
|  | Temporal Fusiform Cortex, posterior division |
|  | Planum Polare |
|  | Heschl's Gyrus |
|  | Planum Temporale |
|  | Hippocampus |

Table S2. Individual results

| **ACC patients** | **language z-scores** | | | **LI** |
| --- | --- | --- | --- | --- |
|  | **language comprehension** | **naming** | **verbal fluency** |  |
| 1 | -.28 | -3.09 | -1.75 | -.09 |
| 2 | -.81 | -3.09 | -2.05 | -.50 |
| 3 | .81 | 1.28 | .10 | .32 |
| 4 | .20 | 1.64 | -.20 | .40 |
| 5 | -.28 | 1.64 | -.95 | .60 |
| 6 | -.47 | -1.75 | -3.09 | .59 |


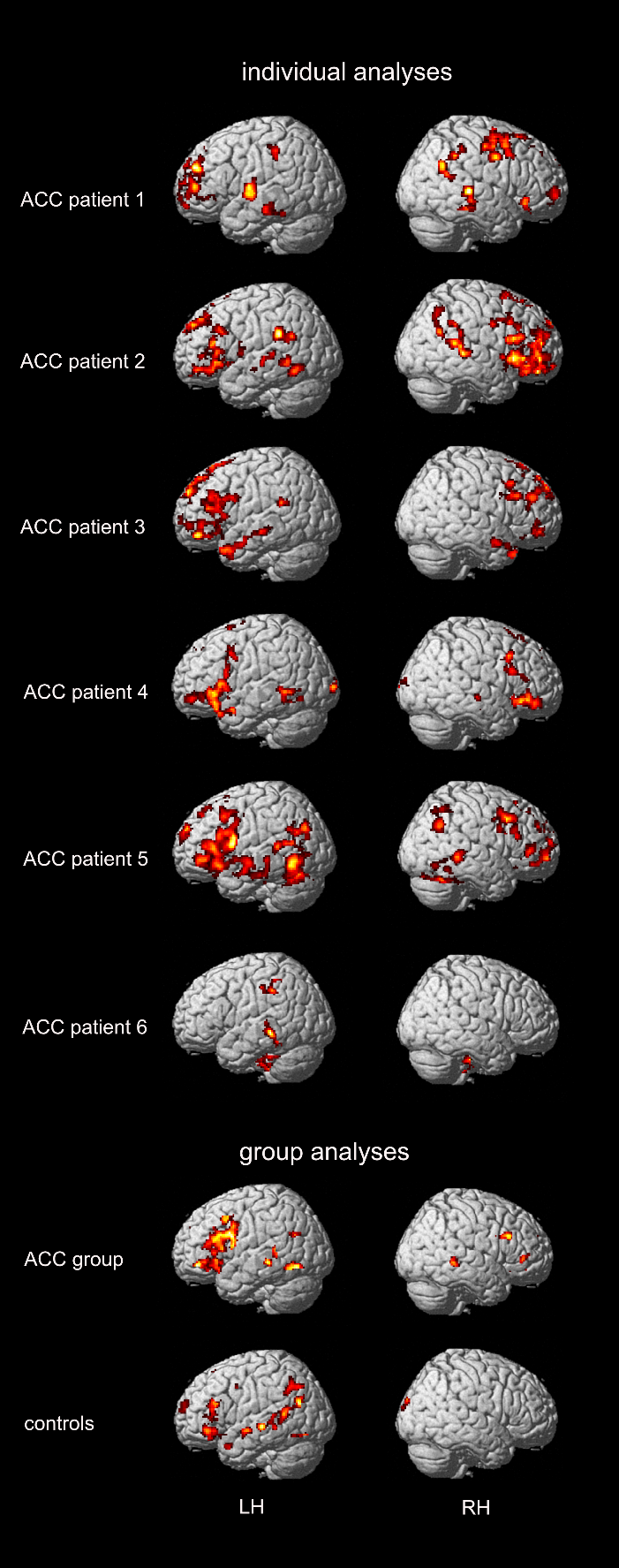


Figure SI. Individual (p_uncorr_ = <.001) and group (fixed effect, p_FWE_ <.05) language activations. Left ist left-hemisphere (LH).
